# High expression of PKM2 synergizes with PD-L1 in tumor cells and immune cells to predict worse survival in human lung adenocarcinoma

**DOI:** 10.7150/jca.42610

**Published:** 2020-05-18

**Authors:** Long Long, Mengxi Chen, Yu Yuan, Alex Lau Ming, Wei Guo, Kaisong Wu, Honglei Chen

**Affiliations:** 1 Department of Pathology, Zhongnan Hospital of Wuhan University, Wuhan 430071, P. R. China; 2 Department of Pathology, School of Basic Medical Sciences, Wuhan University, Wuhan 430071, P. R. China; 3 Department of Respiratory Medicine, Zhongnan Hospital of Wuhan University, Wuhan 430071, P. R. China

**Keywords:** PKM2, PD-L1, Lung adenocarcinoma, Energy metabolism, Immune checkpoint therapy

## Abstract

**Background**: Immunotherapy targeting PD-1/PD-L1 represents a breakthrough in the treatment of lung cancer. Pyruvate kinase M2 (PKM2) is not only a critical player in glycolysis, but also conducive to tumor progression and immune response. While both have been linked to lung adenocarcinoma (AC), the correlation and clinical significance of PKM2 and PD-L1 expression in human lung AC tissues remains not entirely explored.

**Methods**: Expression of PKM2 and PD-L1 proteins were detected by immunohistochemistry in 74 lung AC cases and the corresponding noncancerous tissues. Simultaneously, multiplex immunofluorescence was used to detect PKM2, PD-L1, CK, CD3, and CD68 in the lung AC tissues. We measured expression patterns and co-localization of these markers, evaluating their association with clinicopathological features and overall survival. Validation of findings was conducted using mRNA expression data from The Cancer Genome Atlas (TCGA) of 515 lung AC cases.

**Results**: High expression of PKM2 in tumor cells was significantly related with lymph node metastasis and TNM stage (p=0.035, p=0.017, respectively). Moreover, PKM2 expression in tumor cells was positively correlated with tumor PD-L1 expression. High expression of PKM2, PD-L1 in tumor cells and immune cells predicted high mortality rate and poorer survival rates, respectively. Additionally, multivariate Cox regression models indicated that high expression of PKM2 in tumor cells was an independent prognostic factor. Based on TCGA genomic data, high PKM2 mRNA expression was significantly associated with poorer survival (p=0.001).

**Conclusion**: High expression of PKM2 synergizes with PD-L1 in tumor cells and immune cells to predict poorer survival rates in patients with lung AC.

## Introduction

Lung cancer has long been established as one of the most prevalent and deadly malignant cancer globally 1. It can be subdivided into two types: small cell lung cancer (SCLC) and non-small cell lung cancer (NSCLC). Clinically, NSCLC is the predominant subtype, making up 85% of diagnosed cases. The most frequent subtype of NSCLC is lung adenocarcinoma (AC) 2. Despite the considerable progress of neoadjuvant chemoradiotherapy and molecular targeted therapy, the 5-year overall survival of lung cancer remains poor (18%). Poor survival rates are largely due to local recurrence and distant metastasis 3, 4. As such, it is imperative to identify novel treatment strategies to improve the prognosis of lung AC patients.

Immunotherapy targeting immune checkpoints has been a breakthrough in the treatment of lung cancer 5. Immune checkpoints are regulatory molecules to maintain immune homeostasis, including programmed cell death-1 (PD-1)/programmed cell death ligand-1 (PD-L1), cytotoxic T-lymphocyte antigen-4 (CTLA-4), lymphocyte activation gene-3 (LAG-3), and V-domain Ig suppressor of T cell activation (VISTA), which can collectively inhibit T-cell mediated immune response 6. The inhibitory effects facilitate tumor progression in a process known as “immunoediting”, which has become popular in efforts to enhance anti-tumor immunotherapy 6. PD-1 receptor and the ligands PD-L1 have been shown to be crucial players of immune evasion in cancer 7. With impressive efficacy in clinical trials, PD-1/PD-L1 inhibitors have been approved by the US Food and Drug Administration to treat NSCLC and other cancers 8, 9.

PD-L1 belongs to the B7 family checkpoints, and it is primarily expressed on tumor cells and tumor infiltrating immune cells 10. PD-L1 expression can be regulated by transcriptional factors, such as signal transducer and activator of transcription 3 (STAT3) and nuclear factor-κB (NF-κB) 11. Additionally, hypoxia-inducible factor-1α (HIF-1α) upregulates PD-L1 in hypoxic tumor microenvironment 12. PD-L1 expression is significantly correlated with patient survival and disease progression in several tumors, such as colorectal cancer 13, hepatocellular carcinoma 14 and pancreatic cancer 15. Diverse studies have found that PD-L1 expression can be a plausible predictive biomarker of anti-PD-1/PD-L1 immunotherapy 8. Nevertheless, the response rate of anti-PD-1/PD-L1 immunotherapy remains unsatisfactory, due to tumor resistance and complexity of immune microenvironment 16, 17. Such unsatisfactory response rates call for a deeper understanding of the regulatory mechanisms of PD-L1 expression that will be pivotal for novel combinational immunotherapies, which will in turn, allow more patients to benefit from immunotherapy.

Energy metabolism is an important hallmark of cancer cells, compared with normal cells 18. Cancer cells can shift the oxidative phosphorylation to lactic acid fermentation even under aerobic conditions, which is termed as the Warburg Effect 19. In the final step of glycolysis, pyruvate kinase (PK) acts as the rate-limiting enzyme which can convert phosphoenolpyruvic acid (PEP) into pyruvate 20. There are four PK isozymes, including PKM1, PKM2, PKL, and PKR, whose expression depends on the metabolism of tissues and cells 21. PKM2 is a key player in the genesis and development of tumors, as well as tumoral immune response 22-24. High expression of PKM2 can promote the capacity of glucose uptake, facilitating cell activation and invasion. During the process of metabolic reprogramming, PKM2 regulates glycolytic pathway in activated immune cells and tumor cells 22. In general, PKM2 is mainly present as an inactive dimer or active tetramer, which exhibits different enzymatic properties. Fructose-1,6-bisphosphate (FBP) binding can induce the activation of PKM2, in which PKM2 translocates to the nucleus 25, 26. Additionally, PKM2 serves to be a coactivator of HIF-1α, acting on the nucleus compound consisting of HIF-1α and p300. This interaction relies on proline hydroxylase 3 (PHD3)-mediated hydroxylation, thus modulating diverse proglycolytic enzymes and resulting in cancer progression 27.

At the cellular level, PKM2 is considered to be essential for PD-L1 expression in tumor cells and immunocytes 24. In tumor-mediated hypoxia, activated HIF-1α can simultaneously induce PD-L1 and PKM2 expression, influencing immune evasion and glycolysis 28. However, the relationship and clinical values of PKM2 and PD-L1 proteins in human lung AC tissues remains not entirely explored. Here, we evaluated the association between the critical players of tumor energy metabolism and immune evasion, PKM2 and PD-L1, as well as analyze their correlation with clinicopathological features and overall survival (OS) in 74 lung AC patients. Meanwhile, prognostic value of PKM2 and PD-L1 mRNA was further investigated in 515 cases of lung AC using genomics data from The Cancer Genome Atlas (TCGA). Our research will provide a new insight into the burgeoning field of combinational immunotherapy.

## Materials and Methods

### Patients and follow-up

74 patients diagnosed of lung AC in the period between July 2005 and December 2011 were included in the study. We collected cancerous and corresponding noncancerous tissues, from the Department of Pathology, Zhongnan Hospital of Wuhan University (Hubei, China). Furthermore, 10 specimens had insufficient stroma and the remaining 64 specimens were used to analyze stromal expression. All patients received surgical resection before chemo-radiotherapy or neoadjuvant therapy. The histological diagnosis and grades of differentiation were determined using the 2015 World Health Organization Classification of Lung Tumors 29. All the specimens were reevaluated by two experienced pathologists (Guo W and Chen H) to confirm the histopathologic parameters. Further, 24 patients (32.4%) were subdivided as stage I, 46 (62.2%) as stage II, and 4 (5.4%) as stage III based on the 8th edition of TNM classification by International Union against Cancer (UICC 2017) 30. More detailed clinicopathological features such as gender, age, smoking history, survival status, depth of tumor invasion (T), lymph node metastasis (N), and distant metastasis (M) were described in Table 1.

All procedures involving human participants were in accordance with the ethical standards of the Medical Ethics Committee of Wuhan University Medical College. All research was in compliance with the terms of the 1964 Declaration of Helsinki and its later amendments or comparable ethical standards. Informed consent was obtained from all participants and/or their legal guardian/s.

Follow-up time started after the date of surgery and ended in February 2015. Overall survival (OS) was defined as the interval from initial diagnosis to death or the end of follow-up. Patients, who died of unexpected events or other diseases, were excluded from the survival cohorts. The average overall survival time of lung AC patients was 44 (range: 1-115) months.

### Tissue microarray construction

We constructed two separate lung AC tissue microarrays (TMAs) in this study. The hematoxylin-eosin-stained slides were firstly screened to validate the diagnosis of lung AC by two independent pathologists. The most representative cancerous and noncancerous areas were selected to construct TMA slides. As previously reported 31, one tumor core with a 1.5-mm diameter was taken from every specimen and arranged in paraffin blocks.

### Immunohistochemistry analysis

IHC analysis was performed to detect PKM2 and PD-L1 expression in lung AC tissues. At first, TMA sections were deparaffinized and rehydrated. Subsequently, antigen retrieval of PKM2 was applied in citrate acid buffer (10 mM, pH 6.0) for 15 minutes by microwave. PD-L1 was treated with antigen retrieval in EDTA (1 mM, pH 8.0) buffer by microwave for 20 minutes. The sections were incubated with rabbit anti-human PKM2 polyclonal antibody (1:100 dilution, D78A4, Cell Signaling Technology, USA) and rabbit anti-human PD-L1 polyclonal antibody (1:100 dilution, E1L3N, Cell Signaling Technology, USA) at 4 °C overnight. Then, the TMAs were incubated in horseradish peroxidase (HRP)-conjugated second antibody (Dako REAL EnVision Detection System, Agilent, USA) at 37 °C for 30 minutes, followed by 3,3'-diaminobenzidine (DAB) chromogen (Dako, Agilent, USA) and nuclear counterstaining with hematoxylin.

### Evaluation of immunohistochemistry

Immunostaining reactivity was observed using light microscopy (Olympus BX-53 with CCD DP74). Results were scored by two pathologists (Guo W and Chen H) who were independent and blinded to the clinicopathological characteristics of the study. The scores of the two pathologists were compared, and any discrepancies were reassessed to achieve a consensus.

#### 4.1 Evaluating results of PKM2 expression

PKM2 expression was mainly evaluated in tumor cells and immune cells with the semi-quantitation method in accordance with the area of positive (AP) and the intensity of staining (IS). AP was graded as follows: 0 (0-5%), 1 (6-25%), 2 (26-50%), 3 (51-75%) and 4 (>75%). IS was graded as 0 (negative), 1 (weak), 2 (moderate) and 3 (strong). PKM2 expression was calculated based on the equation: Intensity distribution (ID) = AP × IS 32.

The best cut-off point for PKM2 expression was determined by the receiver operating characteristic (ROC) curve (Supplementary Fig. 1). In accordance with the optimal sensitivity and specificity of the ROC curve by OS, 9.5 was termed as the optimal cut-off point for PKM2 score in tumor cells (≥9.5 = high expression; <9.5 = low expression). Additionally, the optimal cut-off point for PKM2 expression in immune cells was 8.5 (≥8.5 = high expression; <8.5 = low expression).

#### Analyzing results of PD-L1 expression

The expression of PD-L1 protein in tumor cells and immune cells were scored as low or high using 5% as a cut-off point. PD-L1 ≥ 5% was defined as high expression, which was adopted in a number of cancer types 13, 33.

### Multiplex immunofluorescence staining

Manual multiplex immunofluorescence (mIF) staining was performed in 4-μm sections obtained from FFPE lung cancer blocks by using the Opal 7-Color IHC Kit (PerkinElmer, Waltham, MA). The stained slides were scanned by a Vectra multispectral microscope (PerkinElmer) 34. The immunofluorescence markers were consisted of PD-L1 (E1L3N, dilution 1:200; Cell Signaling Technology, USA), PKM-2 (1:200 dilution, D78A4, Cell Signaling Technology, USA), CK(AE1/AE3), CD3(F7.2.38) and CD68 (PG-M1) are ready-to-use antibodies from Agilent/DAKO, California, USA.

Primary antibody was visualized by using tyramide signal amplification linked to a specific fluorochrome from the multiplex IHC Kit for each primary antibody. A stripping procedure, based on the Meidi microwave (Meidi, China), was performed for each consecutive antibody staining. In parallel, uniplex IF was used with each individual antibody and with the same fluorochrome used in the mIF to create the spectral library in human tonsil FFPE tissues used in the multispectral analysis. Human tonsil FFPE tissues were also used with and without primary antibodies as positive and negative (autofluorescence) controls, respectively. The mIF- and uniplex IF-stained slides were scanned with a Vectra 2.0 microscope system (PerkinElmer) under fluorescent illumination. From each slide, Vectra automatically captured the fluorescent spectra from 420 nm to 720 nm at 20-nm intervals with the same exposure time and then combined the captured images to create a single stack image that retained the particulate spectral signature of all IF markers.

### TCGA data analysis for mRNA expression

In order to evaluate the prognosis value of PKM2 and PD-L1, we analyzed the mRNA expression profiles of lung adenocarcinoma (dataset: Tumor Lung Adenocarcinoma - TCGA - 515 - rsem - tcgars) using the R2 genomics analysis and visualization platform (http://r2.amc.nl). This dataset includes 515 lung adenocarcinoma cases. Kaplan-Meier method was applied for survival analysis. The patients with gene expression higher than the 85th percentile of all the patients were grouped to the “high” expression group; the remaining patients were grouped to the “low” expression group. The difference between survival curves of the high expression group and the low expression group was evaluated by log-rank test. Correlation between the PD-L1 and PKM2 was evaluated by Spearman correlation (the data was normalized by calculating the Z scores).

### Statistical analysis

We used SPSS 22.0 software (Chicago, IL, USA) and R 3.3.2 (R Foundation, Vienna, Austria) to perform all statistical analyses. ROC curve analysis was generated to select the best cut-off points for PKM2 protein expression. We explored the relationship between PKM2 and PD-L1 using Spearman correlation analysis appropriately. The χ^2^ test or Fisher exact test were conducted for analyzing the correlation between PKM2 and PD-L1 protein expression with the relevant clinicopathological parameters. Overall survival was estimated using Kaplan-Meier method and log-rank test. We carried out univariate and multivariate Cox proportion hazard regression models of survival to test the independent prognostic impacts. Two-sided *P*-values <0.05 were established as statistically significant.

## Results

### Expression of PKM2 and PD-L1 in lung AC tissues

**Figure 1:**
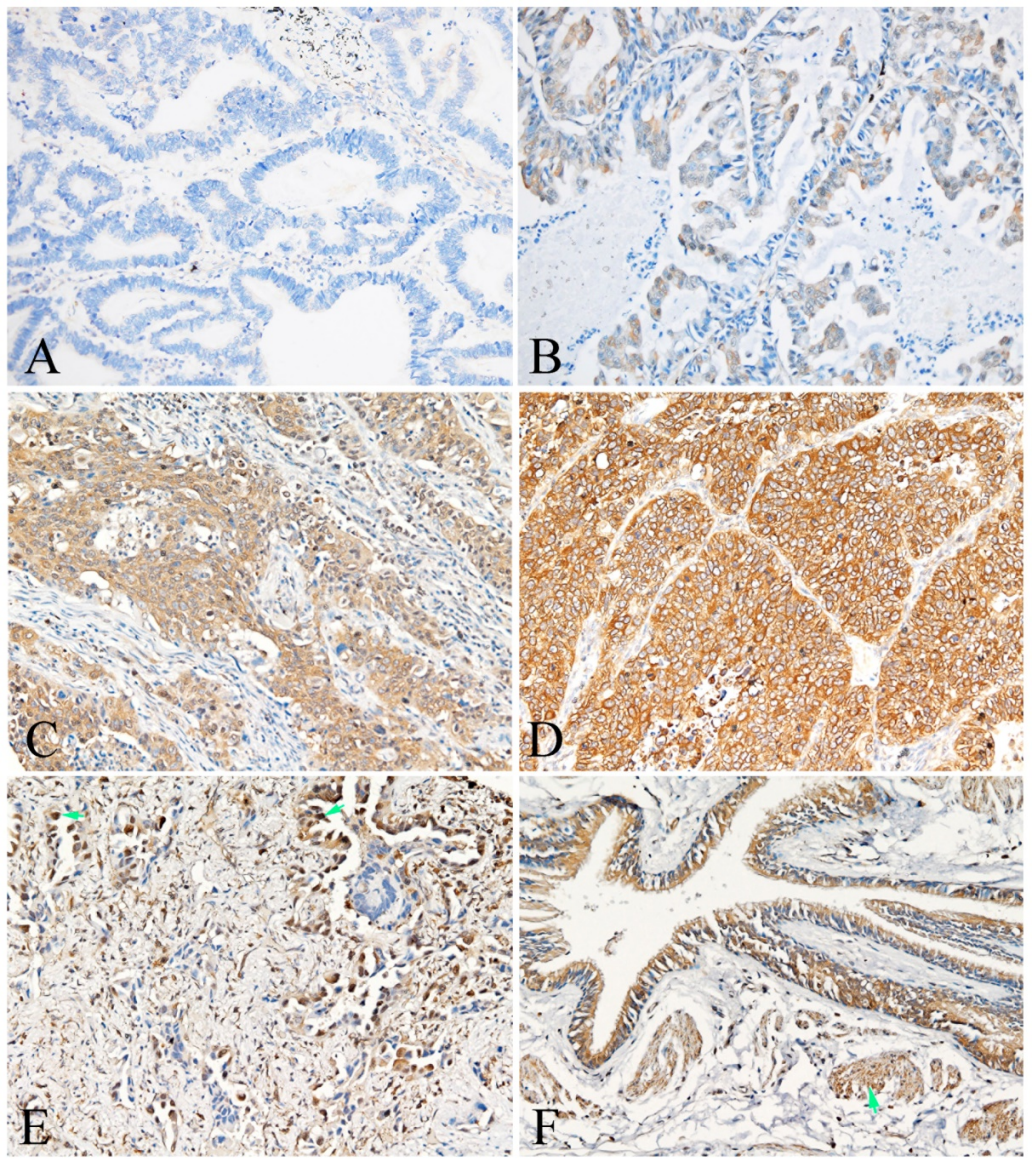
** Representative photomicrographs of PKM2 expression in the lung AC and noncancerous lung tissues.** A showed expression of PKM-2 protein in the lung AC (intensity of staining graded as 0); B showed expression of PKM-2 protein in the lung AC (intensity of staining graded as 1); C showed expression of PKM-2 protein in the lung AC (intensity of staining graded as 2); D showed expression of PKM-2 protein in the lung AC (intensity of staining graded as 3); E showed nuclear PKM-2 protein expression in the lung AC (arrow); F showed positive expression of PKM-2 protein in the bronchial epithelial cells and smooth muscle cells (arrow). (A-F, original magnification ×200).

**Figure 2:**
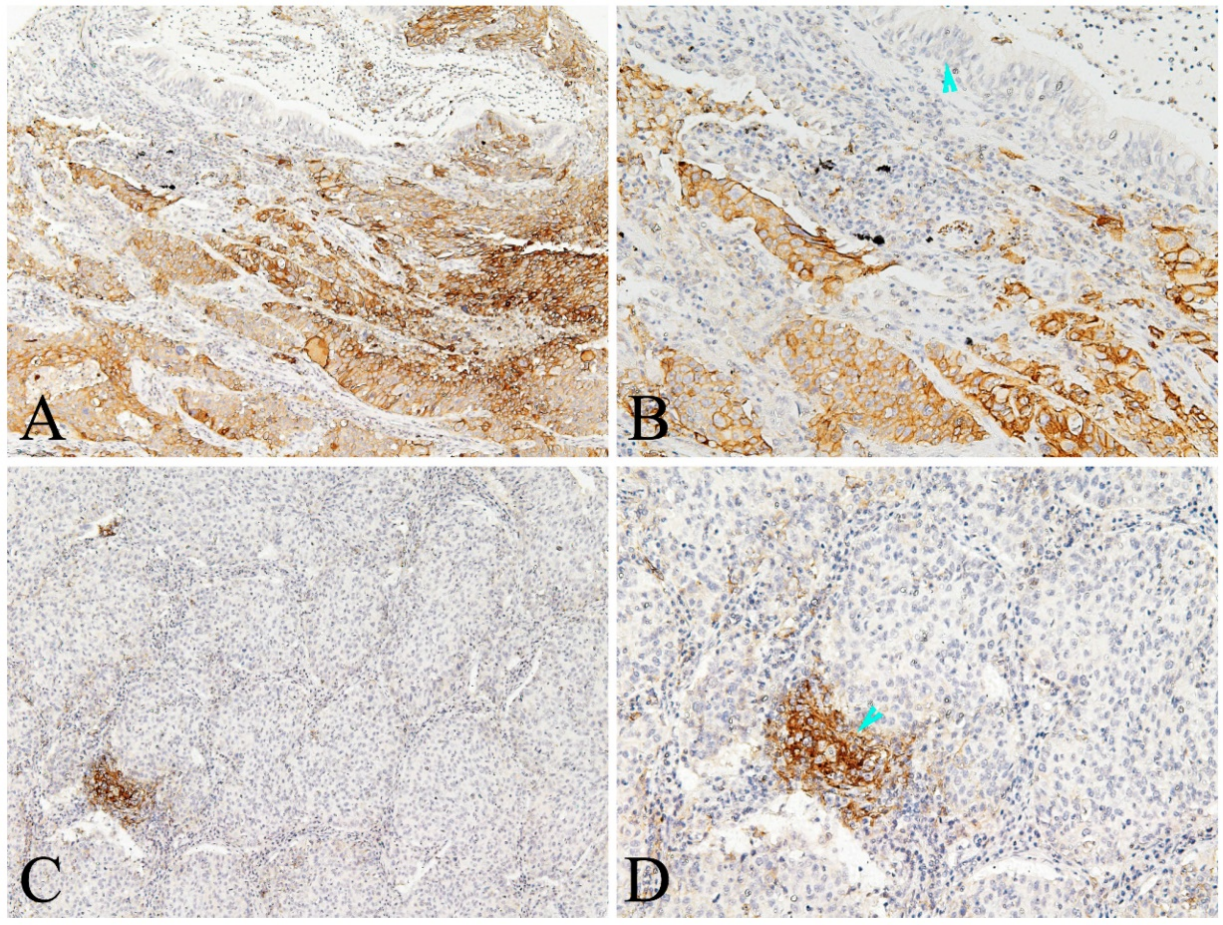
** Representative photomicrographs of PD-L1 expression in the lung AC and noncancerous lung tissues.** A-B showed positive expression of PD-L1 protein in the tumor cells and some immune cells of lung AC, but negative in the paracancerous bronchial epithelial cells (arrowhead); C-D showed positive expression of PD-L1 protein in the immune cells (arrowhead), almost negative in the tumor cells of lung AC (A and C, original magnification ×100; B and D original magnification ×200).

**Figure 3:**
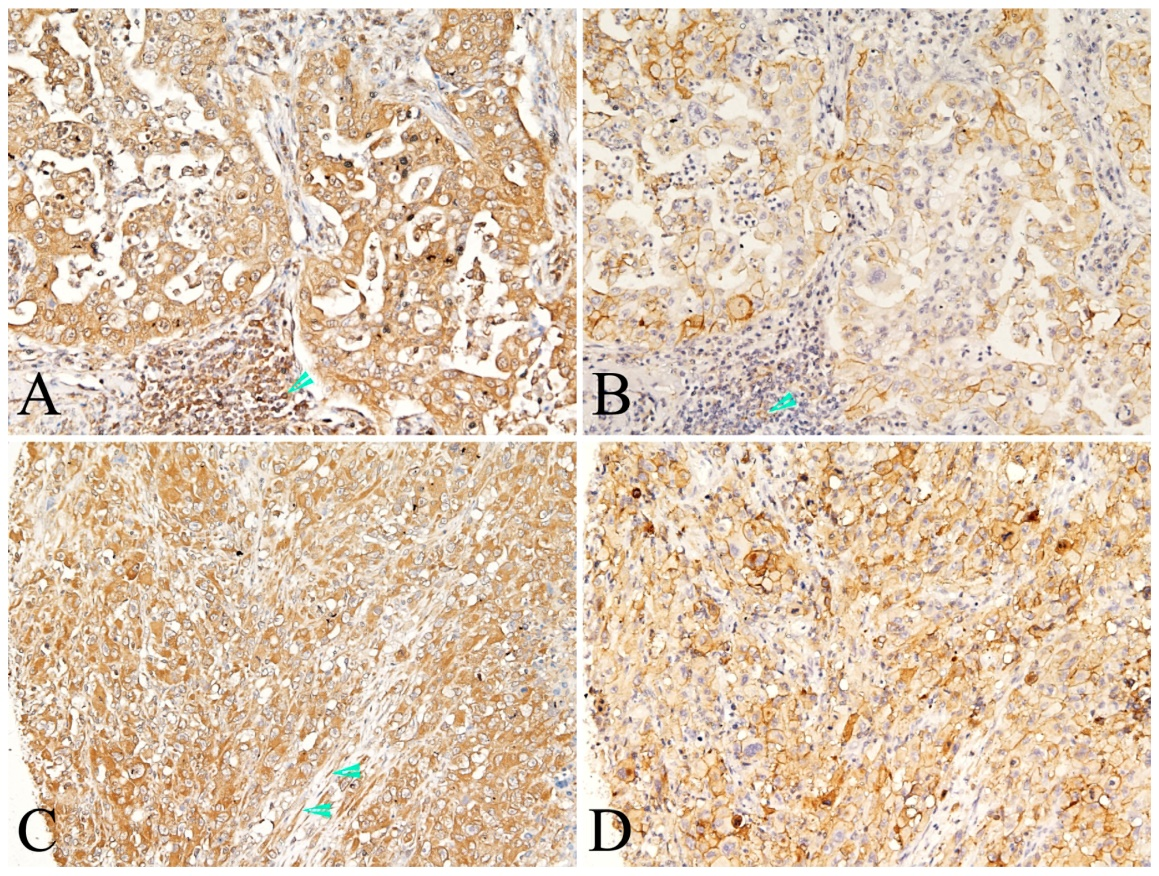
** Representative photomicrographs of co-expression of PKM2 and PD-L1 in the same case of lung AC.** Co-expression of PKM-2(A and C) and PD-L1 (B and D) proteins in the tumor cells of same case, also showed both positive in the immune cells (A and B, arrowhead). Positive expression of PKM-2 also observed in the stromal fibroblasts (C, arrowhead). (A-D, original magnification ×200).

**Figure 4:**
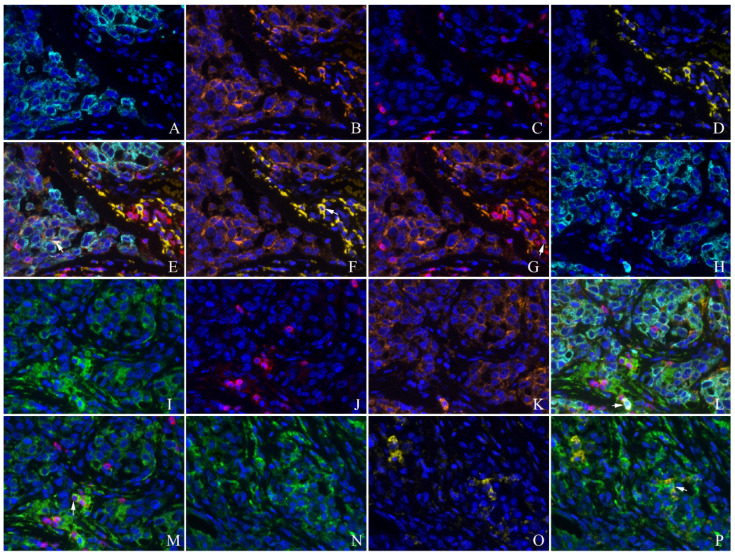
** Co-expression of PKM2 and PD-L1 with CD3, CD68 detected by multiplex immunofluorescence in the lung AC.** A and H: CK(cyan); B and K: PD-L1(orange); C and J:CD3(red); D and O:CD68(yellow); E: unmixed composite image for CK, PD-L1, CD3 and CD68 , co-expression of CK and PD-L1(white arrow showed); F: co-expression of PD-L1 and CD68 (white arrow showed); G: co-expression of PD-L1 and CD3 (white arrow showed); I and N: PKM2(green); L: unmixed composite image for CK, PKM2, PD-L1 and CD3, co-expression of CK, PKM2 and PD-L1(white arrow showed); M: co-expression of PKM2 and CD3 (white arrow showed); P: co-expression of PKM2 and CD68 (white arrow showed) (original magnification of all images ×400).

PKM2 was found to be widely expressed in the majority of human lung AC with distinct spatial patterns. Most of PKM2 was primarily localized in the cell membrane and the cytoplasm of tumor cells (Fig. 1). PKM2 was also stained in the immune cells, fibroblasts, and paracancerous bronchial epithelial cells and smooth muscle cells. Nuclear PKM2 protein was only found in one case (Fig. 1). On the other hand, PD-L1 was mainly detected in the cell membrane of tumor cells, and the cytoplasm of immune cells. However, PD-L1 was not detected in the paracancerous bronchial epithelial cells (Fig. 2). Between the duo, PKM2 signal was generally higher than that of PD-L1, and co-expression of PKM2 and PD-L1 proteins could be observed in some cases of lung AC (Fig. 3 and Fig. 4). From the mIF results, co-expression of PKM2 and CD3, PKM2 and CD68, PD-L1 and CD3, PD-L1 and CD68 were observed, which demonstrated that PKM2 and PD-L1 can both express in the T cells and macrophages (Fig. 4). Based on the cut-offs, PKM2 and PD-L1 exhibited high expression in the lung AC tissues with values of 58.11% (n=43) and 29.73% (n=22), respectively. In the adjacent noncancerous tissues, IHC staining of PKM2 and PD-L1 was significantly lower than that of the cancer tissues.

### Clinicopathological significance of PKM2 and PD-L1 expression in lung AC

We investigated the association between PKM2 and PD-L1 expression and clinicopathological characteristics in lung AC using Chi-squared test. As listed in Table 2a and Table 2b, PKM2 expression in tumor cells was significantly associated with lymph node metastasis (*P*=0.035) and TNM stage (*P*=0.017) in lung AC patients. There was no significant correlation between PD-L1 expression in tumor cells and clinicopathological factors. Meanwhile, co-expression of PKM2 and PD-L1 in tumor cells was significantly related to depth of invasion (*P*=0.027) and TNM stage (*P*=0.029). In contrast, PKM2 expression in immune cells (*P*<0.001) and PD-L1 expression in immune cells (*P*=0.018) were significantly correlated with age. While co-expression of PKM2 and PD-L1 were significant in tumor cells, we did not find any significant correlation between co-expression of PKM2 and PD-L1 in immune cells with clinicopathological factors.

### Relationship between PKM2 and PD-L1 expression in lung AC

Spearman correlation analysis was utilized to examine the relationship between PKM2 and PD-L1 expression in lung AC cohort. 43 patients out of 74 had high PKM2 expression in tumor cells, 14 of those (32.56%) patients showed PD-L1 positivity (Fig. 3). However, out of the remaining 31 patients who had low PKM2 expression in tumor cells, only 8 (25.81%) patients exhibited positive PD-L1 expression. Furthermore, PKM2 expression in tumor cells was positively correlated with tumor PD-L1 expression (r_s_=0.234, *P*=0.045). In the stromal compartment, we found no correlation between PKM2 and PD-L1 protein expression in immune cells (r_s_=0.153, *P*=0.226). However, positive correlation was observed between PKM2 protein and PD-L1 in tumor cells and immune cells (r_s_=0.281, *P*=0.024). There was significant association between PD-L1 in the tumor cells and immune cells (r_s_=0.315, *P*=0.008).

### Prognostic value of PKM2 and PD-L1 in lung AC patients

**Figure 5:**
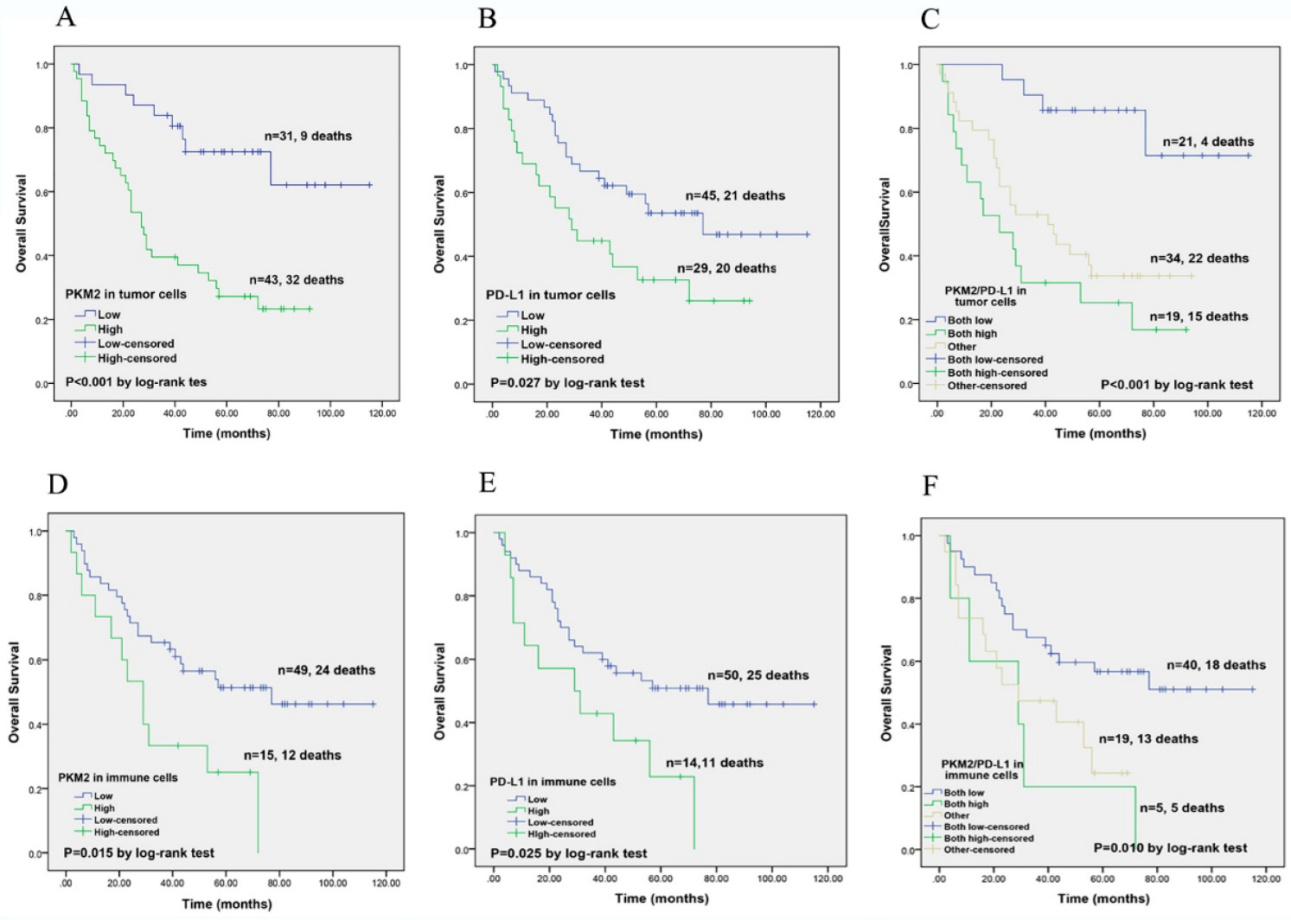
** Kaplan-Meier overall survival analysis of PKM2 and PD-L1 expression in lung AC patients.** (A) In lung AC cohort, high PKM2 expression in tumor cells predicted poorer survival and high mortality rate (*P*< 0.001). (B) High PD-L1 expression in tumor cells showed worse survival compared with the low-expression group (*P*=0.027). (C) Patients with both high PKM2 and PD-L1 expression in tumor cells had the worst OS (*P* < 0.001) than other groups. (D) PKM2 overexpression in immune cells was significantly associated with unfavorable survival (*P*=0.015). (E) Those with high PD-L1 expression in immune cells showed significant correlation with worse overall survival (*P*=0.025). (F) Patients with co-expression of PKM2 and PD-L1 in immune cells predicted poorer survival (*P*=0.010).

Next, the prognostic prediction of PKM2 and PD-L1 was identified by Kaplan-Meier curves and compared using log-rank test. In lung AC cohort, high PKM2 expression in tumor cells predicted poorer survival and high mortality rate (*P*< 0.001). High PD-L1 expression in tumor cells showed worse survival compared with the low-expression group (*P*=0.043). Based on these results, we divided the 74 lung AC patients into three groups for further survival analyses. Patients with both high PKM2 and PD-L1 expression in tumor cells had the worst OS (*P*< 0.001) than other groups (Fig. 5). Meanwhile, high expression of PKM2 in immune cells was significantly associated with unfavorable survival (*P*=0.015), and those with high PD-L1 expression and co-expression of PKM2 and PD-L1 in immune cells showed significant correlation with poorer overall survival (*P*=0.025; *P*=0.010).

We carried out univariate and multivariate analysis of Cox proportional hazard model to analyze the prognostic importance of PKM2, PD-L1 expression and other clinicopathological parameters. In the univariate analysis, PKM2, PD-L1, co-overexpression of both PKM2 and PD-L1 in tumor cells and immune cells, age, and depth of invasion showed significant correlation to the survival of lung AC patients (P<0.001, 0.047, 0.024, 0.019, 0.030, 0.017, 0.037 and 0.021 respectively; Table 3). The independent prognostic value was detected by multivariate analysis. The results identified that PKM2 expression in tumor cells (*P*=0.005, HR: 4.242, 95% CI: 1.550-11.609) was an independent prognosis factor in the overall survival of lung AC cohort (Table 3).

### Clinical implications of PKM2 and PD-L1 mRNA using TCGA data

**Figure 6:**
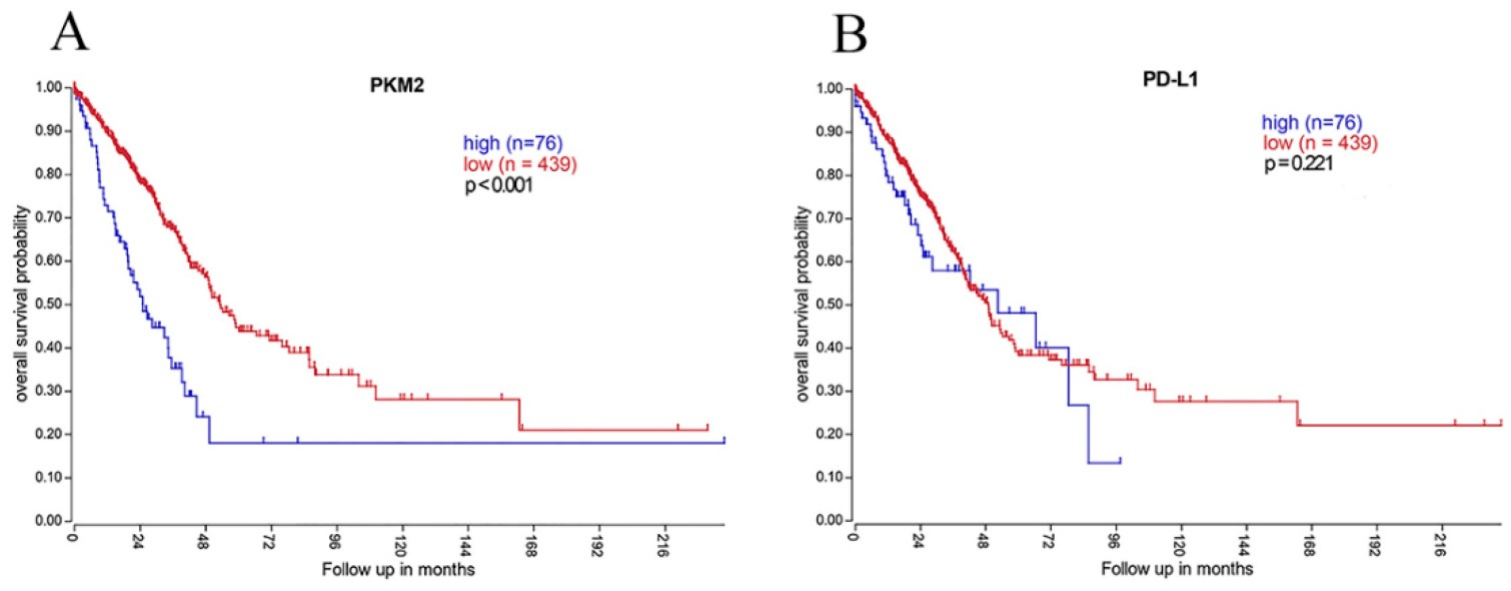
** Kaplan-Meier overall survival analysis of PKM2 and PD-L1 expression using genomics data of lung AC.** (A)In 515 lung AC cases obtained from TCGA dataset, high PKM2 mRNA expression was significantly associated with worse prognosis (*P*< 0.001), which is consistent with our IHC results. (B) However, PD-L1 mRNA expression showed no statistically significant difference between survival curves of the high-expression and low-expression group (*P*=0.221).

To further determine the clinical implications of PKM2 and PD-L1, we analyzed the mRNA expression profiles of 515 lung AC cases. High PKM2 mRNA expression predicted poorer survival and high mortality rate (*P*< 0.001; Fig. 6), which is consistent with our IHC results. However, PD-L1 mRNA expression showed no statistically significant difference between survival curves of the high-expression and low-expression group (*P*=0.221; Fig. 6). Moreover, PKM2 mRNA expression was positively correlated with PD-L1 mRNA expression in comparable TCGA results of lung AC (r_s_=0.126, *P*=0.004).

## Discussion

In recent years, targeting immune checkpoints such as PD-1/PD-L1, has been highlighted as a prominent treatment strategy for lung cancer patients. PD-L1 expression can potentially predict immunotherapy efficacy. However, not all patients respond to PD-1/PD-L1 inhibitors, which poses an urgent need to identify the regulatory mechanism of PD-L1. As a critical player in glycolysis, PKM2 can favor tumor progression and stimulate tumor PD-L1 expression at the cellular level. In this study, we demonstrated that PKM2 and PD-L1 proteins were highly expressed in human lung AC with distinct spatial patterns. We first found that lung AC patients with high expression of both PKM2 and PD-L1 in tumor cells and immune cells had a poorer prognosis. A positive correlation was observed between the expression of PKM2 and PD-L1 in the tumor cells of lung AC tissues.

PKM2 is a major oncogenic factor that regulates tumor progression and cell proliferation. PKM2 plays a crucial role in aerobic glycolysis, which is the predominant metabolic pathway in tumor cells. It correlates with unfavorable survival in hepatocellular carcinoma, melanoma and other tumors 35-37. PKM2 can predict chemotherapy sensitivity in advanced lung cancer patients 38, 39. Moreover, there have been small molecules targeting PKM2 to modulate cellular glucose metabolism 40, 41. In our study, PKM2 was found to be more highly expressed in the majority of lung AC tissues when compared to noncancerous tissues. We have also identified a significant correlation between PKM2 expression and the aggressive development of lung AC, which includes lymph node metastasis and advanced TNM staging. Additionally, there was also a positive correlation between high expression of PKM2 in tumor cells and immune cells, which predicted a poorer clinical outcome in lung AC patients. Furthermore, it was also found that PKM2 expression in the tumor cells could serve as an independent prognostic factor, which indicates that PKM2 is a potential therapeutic target to improve anti-cancer efficacy of lung cancer patients.

Currently, more research is ongoing to characterize the function of PKM2 in tumor immune evasion. PKM2 is considered to be essential for PD-L1 expression 24. As the metabolic enzyme of glycolysis, PKM2 can alter tumor immune microenvironment resulting in low oxygen levels and lactic acid accumulation. A relevant study showed that tumor hypoxic microenvironment due to PKM2 can enhance PD-L1 expression 24. Furthermore, PKM2 facilitates the transactivation of HIF-1α by assembling a nucleus compound of p300, PHD3 and HIF-1α 42, increasing proglycolytic genes in both tumor cells and primary macrophages 27, 43. PKM2 and HIF-1α have a strong interaction with two HRE-binding sites of the PD-L1 promoter in macrophages treated with LPS 24. Our data exhibited that PKM2 is a promising target, maybe to synergize with PD-L1. We also confirmed the positive association between PKM2 and PD-L1 expression in the tumor cells of lung AC tissues. A similar report showed that high expression of both PKM2 and PD-L1 in tumor cells leads to poorer prognosis when compared to other groups 34. On the other hand, we have also found that high expression of PKM2, PD-L1 in immune cells was significantly associated with more unfavorable survival, respectively. Co-expression of PKM2 and PD-L1 in immune cells showed a significant correlation with poorer overall survival. Given this association, it is imperative to figure out the molecular mechanisms of how PKM2 regulates PD-L1 expression.

Despite the remarkable success of PD-1/PD-L1 inhibitors in cancer treatment, not all patients can respond to PD-1/PD-L1 inhibitors, possibly due to the complexity of immune microenvironment and tumor resistance 16. To broaden the spectrum of lung cancer immunotherapy, there is an urgent need to design novel strategies combining immune checkpoint inhibitors plus other treatment modalities. As we mentioned above, PKM2 is a promising therapeutic target of some small molecules under active investigation. Considering the fundamental association between cellular immunology and energy metabolism, we speculate that simultaneously targeting PKM2 and PD-L1 may be a novel strategy to boost the immune system. The patients with co-overexpression of PKM2 and PD-L1 proteins in tumor cells and immune cells may be eligible candidates for combinational immunotherapy. Nevertheless, more rigorous clinical trials are deemed necessary to further our understanding.

Interestingly, PKM2 overexpression predicted unfavorable survival both in protein and mRNA levels while using the TCGA genomics dataset. PD-L1 mRNA expression may be more heterogeneous than PKM2, and demonstrated no prognostic value in RNA sequencing data, which has also been reported in previous research 44. This may be due to the discrepancies between PD-L1 protein and mRNA levels, as some transcription factors are involved in manipulating PD-L1 expression.

Summing up, our findings firstly describe the synergistic effects between PKM2 and PD-L1 expression in both tumor cells and immune cells of human lung AC tissues. Patients with high expression of both PKM2 and PD-L1 in tumor cells and immune cells have a poorer prognosis compared with others. Identifying the novel combination between cellular immunology and energy metabolism may be a hotspot to enhance cancer immunotherapy benefits.

## Supplementary Material

Supplementary figures.

## Figures and Tables

**Table 1:** Patient characteristics in lung AC cohorts.

Characteristics	Sub-characteristics	N (%)
**Age (years)**		59 (32-80)
**Gender**	Male	42 (56.8)
	Female	32 (43.2)
**Survival status**	Death	40 (54.1)
	Survival	34 (45.9)
**Smoking history**	Yes	43 (58.1)
	No	31 (41.9)
**Tumor size (T)**	T1	8 (10.8)
	T2	59 (79.7)
	T3	6 (8.1)
	T4	1 (1.4)
**Lymph node metastasis (N)**	N0	46 (62.2)
	N1	28 (37.8)
	N2	0 (0.0)
**Distant metastasis (M)**	M0	74 (100.0)
	M1	0 (0.0)
**TNM stage**	Ⅰ	24 (32.4)
	Ⅱ	46 (62.2)
	Ⅲ	4 (5.4)
	Ⅳ	0 (0.0)
**Histological type**	Invasive AC	66 (89.2)
	Variant of invasive AC	8 (10.8)
**Total**		74 (100)

**Table 2a:** Associations of PKM2 and PD-L1 protein expression with clinicopathological parameters of lung AC tissues. (a) PKM2 and PD-L1 protein expression in tumor cells.

	PKM2 in tumor cells			PD-L1 in tumor cells			PKM2 and PD-L1		
Parameters	High	Low	*P*	High	Low	*P*	Both high	Others	*P*
**Gender**			0.253			0.197			0.974
Male	22	20		15	27		8	34	
Female	21	11		7	25		6	26	
**Age**			0.235			0.567			0.208
<=64	31	26		16	41		9	48	
>64	12	5		6	11		5	12	
**Depth of invasion (T)**			0.249			0.218			**0.027**
T1,T2	37	30		18	49		10	57	
T3,T4	6	1		4	3		4	3	
**Lymph node metastasis (N)**			**0.035**			0.297			0.075
N0	23	24		12	35		6	41	
N1,N2	20	7		10	17		8	19	
**TNM stage**			**0.017**			0.151			**0.029**
I,II	20	23		10	33		4	39	
III	23	8		12	19		10	21	
**Smoking history**			0.628			0.686			0.603
Yes	17	14		10	21		5	26	
No	26	17		12	31		9	34	
**Histological type**			0.383			0.096			0.339
Invasive	40	26		22	44		14	52	
Variant	3	5		0	8		0	8	

**Table 2b:** Associations of PKM2 and PD-L1 protein expression with clinicopathological parameters of lung AC tissues. (b) PKM2 and PD-L1 protein expression in the immune cells.

	PKM2 in immune cells			PD-L1 in immune cells				PKM2 and PD-L1		
Parameters	High	Low	*P*	High	Low	*P*		Both high	Others	*P*
**Gender**			0.287			0.835				1.000
Male	10	25		8	27			3	32	
Female	5	24		6	23			2	27	
**Age**			**<0.001**			**0.018**				0.575
<=64	4	47		8	43			3	48	
>64	11	2		6	7			2	11	
**Depth of invasion (T)**			0.565			1.000				1.000
T1,T2	15	45		13	47			5	55	
T3,T4	0	4		1	3			0	4	
**Lymph node metastasis (N)**			0.683			0.905				1.000
N0	11	31		9	33			3	39	
N1,N2	4	18		5	17			2	20	
**TNM stage**			0.338			0.847				1.000
I,II	11	27		8	30			3	35	
III	4	22		6	20			2	24	
**Smoking history**			0.688			0.503				0.713
Yes	7	20		7	20			3	24	
No	8	29		7	30			2	35	
**Histological type**			0.738			0.819				1.000
Invasive	14	42		13	43			5	51	
Variant	1	7		1	7			0	8	
										

**Table 3:** COX proportional hazard models on overall survival of lung AC patients.

Factors	Univariate analysis		Multivariate analysis	
	*P* value	HR (95%CI)	*P* value	HR (95%CI)
**Gender**				
Male vs. Female	0.968	1.013 (0.544-1.887)	0.275	1.644 (0.673-4.015)
Age				
<= 64vs. >64	**0.037**	**1.998 (1.041-3.834)**	0.309	1.453 (0.708-2.984)
**PKM2 expression in tumor cells**				
Low vs. High	**<0.001**	**3.772 (1.794-7.930)**	**0.005**	**4.242 (1.550-11.609)**
**PD-L1 expression in tumor cells**				
Low vs. High	**0.047**	**1.909 (1.008-3.615)**	0.052	2.148 (0.992-4.651)
**PKM2 and PD-L1 co-expression in tumor cells**				
Both low vs. both high vs. other				
	**0.024**	**1.519 (1.058-2.181)**	0.716	1.113 (0.626-1.976)
**PKM2 expression in immune cells**				
Low vs. High	**0.019**	**2.320 (1.148-4.689)**	-	-
**PD-L1 expression in immune cells**				
Low vs. High	**0.030**	**2.218 (1.081-4.554)**	-	-
**PKM2 and PD-L1 co-expression in immune cells**				
Both low vs. both high vs. other				
	**0.017**	**1.536 (1.081-2.182)**	-	-
**Depth of invasion(T)**				
T1,T2 vs. T3,T4	**0.021**	**2.685 (1.163-6.198)**	0.578	1.539 (0.337-7.020)
**Lymph node metastasis(N)**				
N0 vs. N1,N2	0.233	1.456 (0.785-2.703)	0.381	2.241 (0.368-13.638)
**TNM stage**				
I,II vs. III	0.053	1.836 (0.993-3.395)	0.297	0.331 (0.042-2.639)
**Smoking history**				
Yes vs. No	0.537	0.820 (0.437-1.539)	0.607	0.782 (0.307-1.991)
**Histological type**				
Invasive vs. Variant	0.303	1.578 (0.662-3.759)	0.691	1.172 (0.535-2.567)
