# Supplementary material for: High expression of PKM2 synergizes with PD-L1 in tumor cells and immune cells to predict worse survival in human lung adenocarcinoma
Source: J Cancer. 2020 May 18;11(15):4442–52. doi: 10.7150/jca.42610 (PMC7255362; doi:10.7150/jca.42610)
Supplement: Supplementary file 1 — Supplementary figures. [file jcav11p4442s1.pdf]

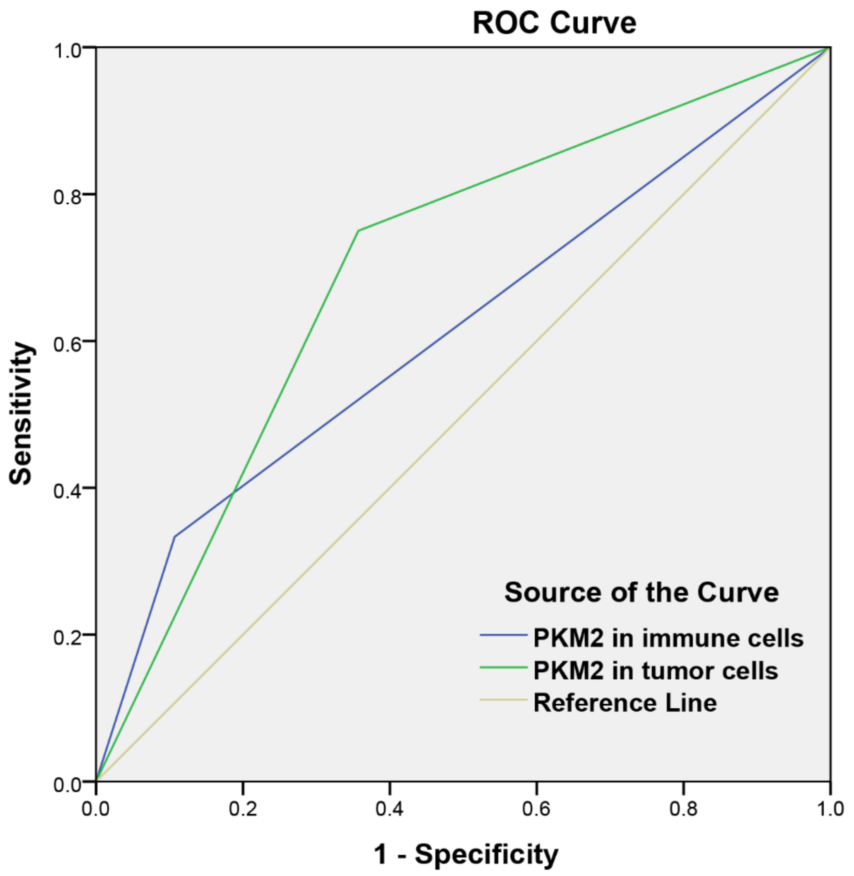

Figure S1. ROC analysis of PKM2 protein by overall survival in lung AC patients.

The cut-offs of PKM2 in tumor cells (≥9.5 = high expression; <9.5 = low expression) and immune cells (≥8.5 = high expression; <8.5 = low expression) have optimal sensitivity and specificity. PKM2 expression level in tumor cells (area under the curve was 0.696, 95% CI: 0.563-0.829; *P* = 0.007). PKM2 expression level in immune cells (area under the curve was 0.613, 95% CI: 0.475-0.751; *P* = 0.123).
